# Cyclic Hydroxylamines as Monitors of Peroxynitrite and Superoxide-Revisited

**DOI:** 10.3390/antiox11010040

**Published:** 2021-12-24

**Authors:** Uri Samuni, Amram Samuni, Sara Goldstein

**Affiliations:** 1 Department of Chemistry & Biochemistry, Queens College, City University of New York, Flushing, NY 11367, USA; Uri.Samuni@qc.cuny.edu; 2 The PhD Programs in Biochemistry and Chemistry, The Graduate Center of the City University of New York, New York, NY 10016, USA; 3 Institute of Medical Research, Israel-Canada Medical School, The Hebrew University of Jerusalem, Jerusalem 91120, Israel; ramisamuni@gmail.com; 4 Institute of Chemistry, The Hebrew University of Jerusalem, Jerusalem 91904, Israel

**Keywords:** spin probe, cyclic nitroxide, hydroxylamine, SOD-mimic, kinetics, EPR

## Abstract

There is a considerable need for methods that allow quantitative determination in vitro and in vivo of transient oxidative species such as peroxynitrite (ONOOH/ONOO^−^) and superoxide (HO_2_^•^/O_2_^•−^). Cyclic hydroxylamines, which upon oxidation yield their respective stable nitroxide radicals, have been suggested as spin probes of peroxynitrite and superoxide. The present study investigated this approach by following the kinetics of peroxynitrite decay in the absence and presence of various 5-membered and 6-membered ring hydroxylamines, and comparing the yield of their respective nitroxides using electron paramagnetic spectroscopy. The results demonstrate that hydroxylamines do not react directly with peroxynitrite, but are oxidized to their respective nitroxides by the radicals formed during peroxynitrite self-decomposition, namely ^•^OH and ^•^NO_2_. The accumulated nitroxides are far below their expected yield, had the hydroxylamines fully scavenged all these radicals, due to multiple competing reactions of the oxidized forms of the hydroxylamines with ^•^NO_2_ and ONOO^−^. Therefore, cyclic hydroxylamines cannot be used for quantitative assay of peroxynitrite in vitro. The situation is even more complex in vivo where ^•^OH and ^•^NO_2_ are formed also via other oxidizing reactions systems. The present study also compared the yield of accumulated nitroxides under constant flux of superoxide in the presence of various cyclic hydroxylamines. It is demonstrated that certain 5-membered ring hydroxylamines, which their respective nitroxides are poor SOD-mimics, might be considered as stoichiometric monitors of superoxide in vitro at highest possible concentrations and pH.

## 1. Introduction

Superoxide (HO_2_^•^/O_2_^•−^) is formed during normal metabolism, as well as in pathophysiological processes through the action of various drugs, poisons and radiation [1]. Superoxide is a moderately reactive radical that oxidizes relatively few biological compounds [2]. However, there are some reactions of superoxide that contribute to its toxicity and are potentially deleterious including its recombination with NO thus forming peroxynitrite (ONOOH/ONOO^−^), which is implicated in pathophysiology of various diseases including acute and chronic inflammatory processes, sepsis, ischemia-reperfusion, and neurodegenerative disorders [3,4].

**Scheme 1:**
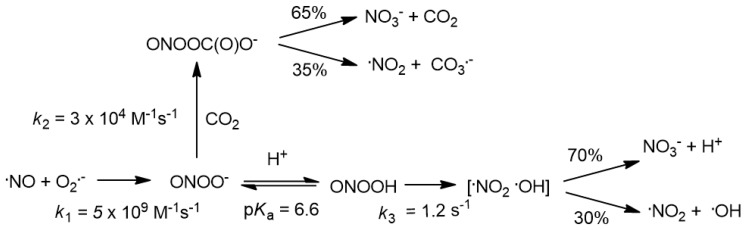
Mechanism of peroxynitrite formation and decomposition in the absence and presence of CO_2_ at 25 °C.

Numerous methods for determination of superoxide in biological systems have been published like the use of fluorescent and chemiluminescent probes, spectrophotometry and spectrometry methods, as well as chromatography and genetically encoded fluorescent protein-based assays. All have their advantages and disadvantages [5,6]. Similarly, the determination of peroxynitrite, particularly in biological systems, has been a challenge. It requires detector molecules that can efficiently outcompete the multiple reactions that peroxynitrite can undergo. A peroxynitrite detector could potentially enable studies that discriminate its biological effects from those of its precursors, NO and O_2_^•−^, and its radical products, ^•^OH, CO_3_^•−^ and ^•^NO_2_ (Scheme 1) [7].

Furthermore, assays for peroxynitrite that are based on detection of its transient radical products are not valid because these radicals are formed also by other oxidizing reactions systems such as ionizing radiation, UV/H_2_O_2_ and peroxidase/H_2_O_2_/nitrite [8].

One of the methods suggested for a quantitative determination of superoxide and peroxynitrite makes use of cell-permeable cyclic hydroxylamine, such as 1-hydroxy-2,2,6,6-tetramethyl-4-oxo-piperidine (TEMPONE-H) and 1-hydroxy-3-carboxy-2,2,5,5-tetramethyl-pyrrolidine. This approach assumes efficient oxidation of cyclic hydroxylamines by superoxide and peroxynitrite to produce stable nitroxide radicals as end-products, which can be quantitatively determined by electron paramagnetic resonance (EPR) spectroscopy [6]. The rate constants of their reactions with superoxide and peroxynitrite have been previously reported to be 10^3^–10^4^ M^−1^ s^−1^ [9,10,11] and >10^9^ M^−1^ s^−1^ [9,10,12], respectively. The present study demonstrates that cyclic hydroxylamines (RNO-H) do not react directly with peroxynitrite, but rather indirectly with the radicals formed during its self-decomposition (Scheme 1). In addition, due to multiple competing reactions of the oxidized forms of RNO-H, i.e., nitroxide (RNO**^•^**) and oxoammonium cation (RN^+^=O), with ^•^NO_2_ and ONOO^−^, and the formation of ^•^OH and ^•^NO_2_ by other oxidizing systems [8,13], the determination of [RNO^•^] thus accumulated does not validly reflect the formation of peroxynitrite and cannot be used for its quantitative or even qualitative determination.

Previously, we used xanthine/xanthine oxidase as a generating system of superoxide at a constant rate, and observed that scavenging of superoxide by TEMPO-H and TEMPOL-H (the reduced forms of 2,2,6,6-tetramethyl-piperidine-1-oxyl and 4-hydroxy-2,2,6,6-tetramethyl-piperidine-1-oxyl respectively) decreased progressively with time [11]. Also, the efficacy of superoxide scavenging by these hydroxylamine increases upon increasing the pH [11]. The scavenging of superoxide by the hydroxylamines is accompanied by accumulation of their respective nitroxides, which effectively catalyze the dismutation of superoxide (frequently coined SOD-mimics or having SOD-like activity) and outcompetes the scavenging of superoxide by the hydroxylamine. The SOD-like activity of these nitroxides decreases as the pH increases [14,15] resulting in less effective competition for superoxide, thus explaining the higher efficacy of superoxide scavenging by the hydroxylamines at higher pH [11]. The present study demonstrates that hydroxylamines, which upon oxidation yield nitroxides that, are poor SOD-mimics, e.g., 5-membered ring nitroxides [15], might be considered and tested under specific experimental conditions as stoichiometric monitors of superoxide.

## 2. Materials and Methods

### 2.1. Materials

Water for preparation of the solutions was purified using a Milli-Q purification system. All chemicals were of analytical grade and were used as received. The following products were purchased from Sigma-Aldrich: 2,2,6,6-tetramethyl-piperidine-1-oxyl (TEMPO, TPO), 4-OH-TPO (TEMPOL), 4-oxo-TPO (TEMPONE), 3-carbamoyl proxyl (3-CP), 3-carbamoyl-2,2,5,5-tetramethyl-3-pyrrolin-1-oxyl (3-CTPO), superoxide dismutase from bovine erythrocytes (SOD), cytochrome c Type VI from horse heart (Cyt^III^) and diethylenetriaminepentaacetic acid (DTPA).

**Scheme 2:**
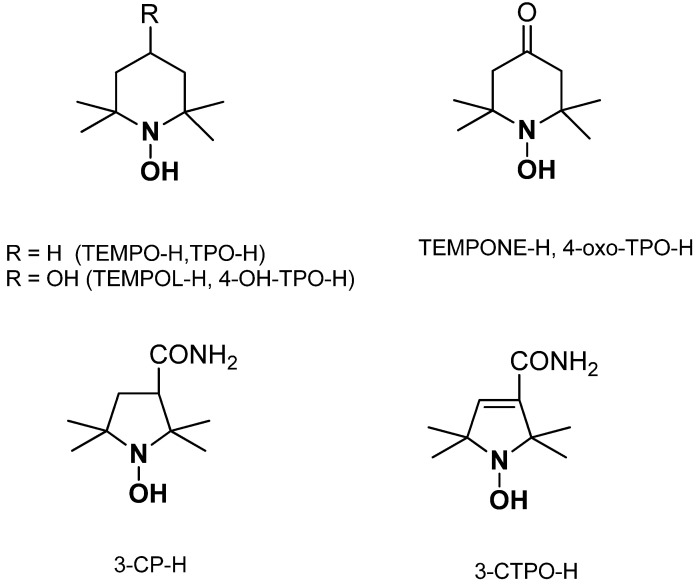
Structures of RNO-H studied.

Solutions of RNO-H were prepared by bubbling H_2_ gas through aqueous solutions containing 0.05–0.2 M RNO^•^ in the presence of an excess HCl and Pt powder for at least 30 min. Bubbling of H_2_ ceased when the residual RNO^•^ decrease to less than 0.2% of its starting concentration as determined by EPR spectroscopy. This residual concentration was even lower when using hydrochloride salt of TEMPOL-H that was prepared by bubbling HCl gas through ethanolic solution of the nitroxides followed by drying, i.e., <0.1%. The concentration of RNO-H was determined after dilution in 0.1 M phosphate buffer (pH 8) and re-oxidation to RNO^•^ by ferricyanide followed by EPR spectroscopy. Scheme 2 displays the structures of RNO-H studied.

Peroxynitrite was synthesized through the reaction of nitrite with acidified H_2_O_2_ using a quenched flow with a computerized syringe pump (“World Precision Instruments” Model SP 230IW) as previously described [16]. Briefly, 0.6 M H_2_O_2_ in 0.7 M HClO_4_ was mixed with 0.6 M nitrite and the mixture was quenched with 3.6 M NaOH at a flow rate of 45 mL/min. The yield of peroxynitrite was determined by measuring the absorbance at 302 nm using ε_302_ = 1670 M^−1^ cm^−1^.

### 2.2. Methods

#### 2.2.1. Rapid-Mixing Stopped-Flow

Kinetic measurements were carried out using the Bio SX-17MV Sequential Rapid-mixing Stopped-Flow apparatus from Applied Photophysics equipped with a 1-cm optical path. All experiments were carried out at 25 °C. At least three independent experiments were performed, and the rate constant given represents an average of 3–5 measurements for each substrate concentration or pH.

#### 2.2.2. Electron Paramagnetic Resonance (EPR)

EPR spectra were recorded at room temperature using a Varian E4 X-band spectrometer operating at 9.36 GHz with the center field set at 3325 G, 100 kHz modulation frequency, 2 G field modulation amplitude, and 20 mW incident microwave power. Samples of the reaction mixture were injected into a flexible capillary, which was inserted into a quartz tube placed within the EPR spectrometer cavity. RNO^•^ concentrations were calculated from the EPR signal intensity using standard solutions of RNO^•^. The contamination of RNO-H solutions by RNO^•^ was determined immediately before each measurement using EPR spectroscopy. RNO-H concentration was determined from the EPR signal intensity of RNO^•^ obtained upon dilution with 0.1 M phosphate buffer (pH 8) and oxidation of RNO-H by 2 mM ferricyanide. In this case, the calibration curve was prepared with standard solutions of RNO^•^ containing 2 mM ferricyanide.

#### 2.2.3. Continuous Radiolysis

Steady-state γ-irradiation experiments were carried out at room temperature using a ^137^Cs source. The dose rate was determined to be 6.5 Gy min^−1^ using Fricke dosimetry using G(Fe^III^) = 15.6 and ε(Fe^III^)_302_ = 2200 M^−1^ cm^−1^.

#### 2.2.4. Determination of the Rate Constant of Superoxide Reaction with Hydroxylamine

Superoxide was radiolytically generated in aerated or oxygenated solutions containing formate, phosphate buffer and 50 μM DTPA to bind all traces of redox-active transition metals thus avoiding metal-catalyzed dismutation of superoxide. Under such experimental conditions all radicals produced by the radiation are converted into superoxide [2]. The rate constant of hydroxylamine reaction with superoxide (*k*_RNO-H_) was determined using oxygenated solutions containing 1 mM hydroxylamine, 0.1 M formate and 10 mM phosphate buffer at pH 7.8. A competition between hydroxylamine and SOD for superoxide yields Equation (4) where [RNO**^•^**]_0_ denotes the accumulated concentration of nitroxide in the absence of SOD.

[RNO^•^]_0_/[RNO^•^] = 1 + *k*_cat_[SOD]/*k*_RNO-H_[RNO-H]_0_ (4)

A plot of ([RNO^•^]_0_/[RNO^•^]-1) vs. [SOD] yields a straight line and one has to determine *k*_cat_ to calculate *k*_RNO-H_. As previously described, *k*_cat_ = (2.8 ± 0.2) × 10^9^ M^−1^ s^−1^ was determined using Cyt^III^ as a competing reagent in aerated solutions containing 20 μM Cyt^III^, 5 mM formate and 1 mM phosphate buffer at pH 7.8 where *k*(Cyt^III^ + O_2_^•−^) = 1.1 × 10^6^ M^−1^ s^−1^ [17]. However, *k*_cat_ is highly affected by ionic strength and in the presence of 0.1 M formate it decreases to *k*_cat_ = (1.3 ± 0.1) × 10^9^ M^−1^ s^−1^ [18].

## 3. Results

### 3.1. Reaction of Peroxynitrite with Hydroxylamines

**Scheme 3:**
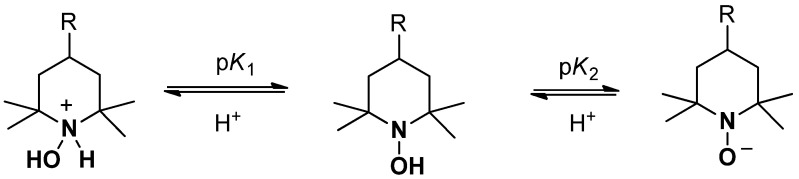
Different forms of hydroxylamine, RN^+^HOH, RNO-H, RNO^−^.

The p*K*_a_ of ONOOH, 6.5–6.8 [19,20,21], significantly increases in the presence of high concentrations of buffers, e.g., 8.59 in the presence of 0.1 M borate [21]. Hydroxylamines are weak bases and have two p*K*_a_ values as demonstrated for the 6-membered ring in Scheme 3.

The value of p*K*_1_ varies between 4 and 8, e.g., p*K*_1_(3-CTPO-H) = 4.3 [22], p*K*_1_(3-CP-H) = 5.85 [22], p*K*_1_(TEMPOL-H) = 7.1 [23] and p*K*_1_(TEMPO-H) = 7.5 [23], 7.96 [22]. The value of p*K*_2_ is significantly higher, e.g., 13.7 has been estimated for TEMPOL-H and TEMPO-H [23]. Therefore, under our experimental conditions the predominant forms are RNO-H and RN^+^HOH.

#### 3.1.1. Reaction of RN^+^HOH with ONOOH

**Figure 1:**
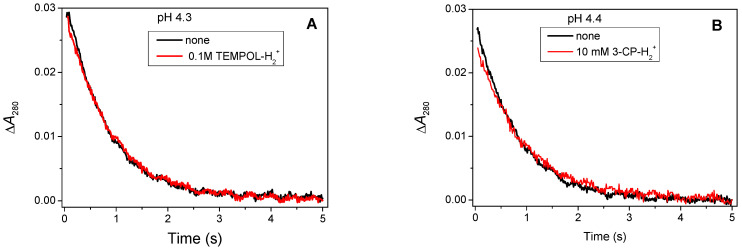
Reaction of ONOOH with RN^+^HOH. Absorbance changes (Δ*A*_280_) observed when (**A**) 135 μM peroxynitrite decayed at pH 4.3 in the absence (black curve) and presence of 0.1 M TEMPOL-H_2_^+^ (red curve); (**B**) 120 μM peroxynitrite decayed at pH 4.4 in the absence (black curve) and presence of 10 mM 3-CP-H_2_^+^ (red curve). Solutions contained 50 mM acetate buffer and 50 μM DTPA.

Aerated solutions containing hydroxylamine in 0.1 M acetate buffer and 100 µM DTPA were mixed using the rapid-mixing stopped-flow at a 1:1 volume ratio with aerated solution containing 240–270 µM ONOO^−^ and 10 mM NaOH. The final pH as measured at the outlet of the stopped-flow apparatus was 4.3–4.4. Therefore, the predominant form of the hydroxylamine under such experimental conditions is RN^+^HOH. Although the maximum absorption of ONOOH is around 250 nm [20], its decay was followed at 280 nm to avoid/reduce interfering absorption due to RN^+^HOH absorption in this spectral region. In the absence of RN^+^HOH the decay of ONOOH followed first-order kinetics where *k*_d_ = 1.32 ± 0.03 s^−1^, in agreement with literature data [7]. The decay of ONOOH was unaffected by the presence of either 5–100 mM TEMPOL-H_2_^+^, 1–5 mM TEMPO-H_2_^+^, or 2.5–10 mM 3-CP-H_2_^+^ (e.g., Figure 1).

In the presence of 0.625 mM and 1.25 mM TEMPONE-H_2_^+^, *k*_d_ increased somewhat to 1.43 ± 0.03 s^−1^ and 1.63 ± 0.09 s^−1^, respectively. Further increase of [TEMPONE-H_2_^+^] to 2.5 mM had no effect on the rate, i.e., *k*_d_ = 1.58 ± 0.07 s^−1^.

#### 3.1.2. Reaction of RNO-H with ONOO^−^

**Figure 2:**
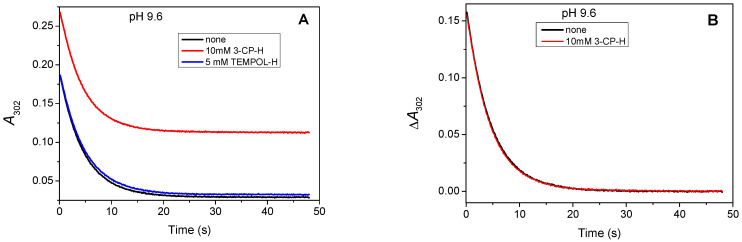
Reaction of ONOO^−^ with RNO-H. (**A**) Decay of 120 μM ONOO**^−^** monitored at 302 nm in the absence (black curve) and in the presence of 10 mM 3-CP-H (red curve) or 5 mM TEMPOL-H (blue curve) at pH 9.6; (**B**) absorbance changes (Δ*A*_302_) in the absence and presence of 10 mM 3-CP-H at pH 9.6. Solutions contained 0.1 M borate buffer and 100 μM DTPA.

Deaerated solutions containing RNO-H in 0.2 M borate buffer and 200 µM DTPA were mixed at a 1:1 volume ratio with aerated solution containing 240–270 µM ONOO^−^ and 10 mM NaOH using a rapid-mixing stopped-flow apparatus. The reaction of CO_2_ with ONOO^−^ is relatively fast (Scheme 1), and therefore, the buffer solutions were bubbled with N_2_ for at least 1 h to remove CO_2_ from the solutions. The final pH as measured at the outlet of the stopped-flow apparatus was 9.6. At this pH the predominant form of the hydroxylamine is RNO-H and under such experimental conditions the rate of the self-decomposition of peroxynitrite, i.e., *k*_d_ = 0.22 ± 0.01 s^−1^, was unaffected by the presence of 5 mM TEMPO-H or 10 mM 3-CP-H (Figure 2).

#### 3.1.3. Reaction of RNO-H/RN^+^HOH with ONOOH/ONOO^−^

The reaction of ONOOH/ONOO^−^ with either TEMPONE-H/TEMPON-H_2_^+^ at pH 7.0 or TEMPOL-H/TEMPOL-H_2_^+^ at pH 7.7 (deaerated buffer solutions, 100 μM DTPA, 0.1 M phosphate buffer). The rate of peroxynitrite decay monitored at 302 nm was unaffected upon increasing the concentration of TEMPONE-H/TEMPON-H_2_^+^ from 0.625 mM to 2.5 mM or TEMPOL-H/TEMPOL-H_2_^+^ from 4 mM to 40 mM.

#### 3.1.4. EPR Measurements

EPR measurements show that ONOOH decomposition in the presence of RN^+^HOH at pH 4.3–4.6 (50 mM acetate buffer and 50 µM DTPA) yields RNO^•^, but its yield was significantly lower than ca. 60% expected if all ^•^OH and ^•^NO_2_ radicals (Scheme 1) stoichiometrically oxidize the hydroxylamine to nitroxide (Table 1).

At pH 7.0–7.7 (0.1 M phosphate buffer and 50 µM DTPA), the yield of RNO**^·^** was also significantly lower than the expected yield of 60%, e.g., 8.6% in the presence of 2.5 mM TEMPONE-H/TEMPONE-H_2_^+^ at pH 7.0 and 20.1% in the presence of 10 mM TEMPOL-H/TEMPOL-H_2_^+^ at pH 7.7.

### 3.2. Reaction of Superoxide with Hydroxylamines

The present study compared the efficacies of superoxide scavenging by 5-membered and 6-membered ring hydroxylamines, where their respective nitroxides have different SOD-mimic activities. Superoxide was radiolitically generated in oxygenated solutions containing 0.1 M formate, 10 mM phosphate buffer and 50 μM DTPA. Under such experimental conditions all radicals produced by the radiation are converted into superoxide (HO_2_^•^/O_2_^•−^, p*K*_a_ = 4.8) [2], and the rate of its formation was 4.0 ± 0.1 μM/min.

TEMPOL-H and TEMPONE-H, even when present at relatively high concentrations, failed to fully scavenge most of superoxide formed (Table 2). However, the catalytic dismutation of superoxide by 5-membered ring nitroxides is about 2-orders of magnitude lower than that by 6-membered ones [14,15], and therefore, 5-membered ring hydroxylamines at sufficiently high concentration and pH fully scavenge superoxide as demonstrated in the case of 3-CP-H and 3-CTPO-H in Table 2. This also explains why contamination of the hydroxylamine solutions by nitroxide had a significant effect only in the case of the 6-membered ring hydroxylamines (Table 2).

**Figure 3:**
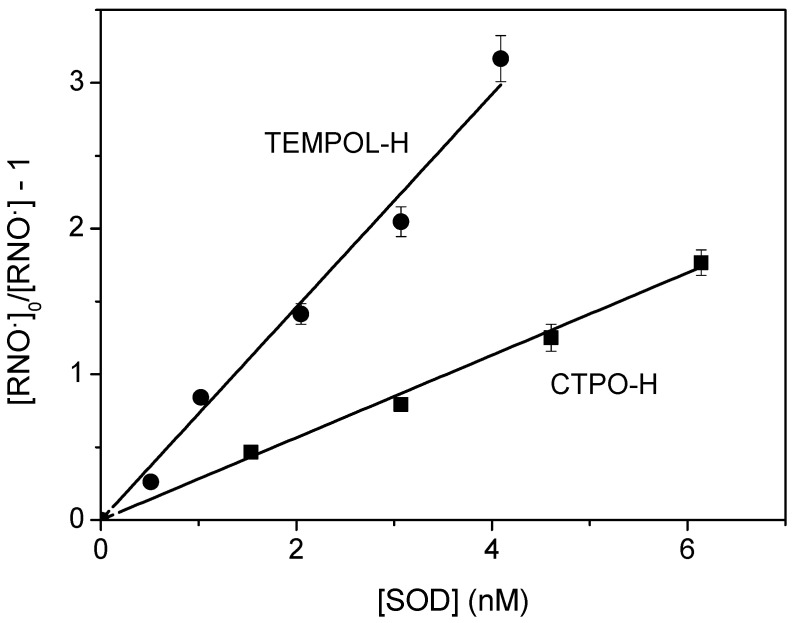
Reaction of superoxide with hydroxylamine. Competition kinetics using SOD as a reference solute. The accumulated [RNO^•^] was monitored upon irradiation of oxygenated solution containing 1 mM CPTO-H (■) or TEMPOL-H (●), 0.1 M formate, 50 μM DTPA, 10 mM PB, pH 7.8, in the absence and in the presence of various concentrations of SOD where [RNO^•^]_0_ denotes the accumulated concentration of nitroxide in the absence of SOD.

The rate constant of 3-CTPO-H reaction with superoxide has been determined to be (4.6 ± 0.2) × 10^3^ M^−1^ s^−1^ at pH 7.8 (0.1 M formate, 10 mM phosphate buffer, 50 μM DTPA) using SOD as a competing agent as described in the experimental section (Figure 3).

Under the same experimental conditions, we determined *k* = (4.7 ± 0.2) × 10^3^ M^−1^ s^1^ for 3-CP-H and (1.8 ± 0.1) × 10^3^ M^−1^ s^−1^ for TEMPOL-H (Figure 3), which are in excellent agreement with the values determined directly by pulse radiolysis [11]. Since the rate constants for the 5-membered ring hydroxylamines are similar, the less efficacy of 3-CP-H most probably due to having respective nitroxide that is a better SOD-mimic [14,15].

## 4. Discussion

### 4.1. Peroxynitrite Reaction with Hydroxylamines

The results demonstrate that RN^+^HOH does not react directly with ONOOH. Similarly, RNO-H does not react directly with ONOO^−^. In fact, the rate of peroxynitrite self-decomposition outcompetes the rate of its direct reaction with hydroxylamine even in the presence of 0.1 M TEMPOL-H. Moreover, peroxynitrite does not react directly with hydroxylamines in the presence of CO_2_ (Scheme 1), which is the predominate path of peroxynitrite decomposition in vivo. The rate constant of peroxynitrite with hydroxylamines has been claimed to be >10^9^ M^−1^ s^−1^ by measuring the yield of the respective nitroxide in the presence of DMSO [9,10,12]. In fact, the competition that took place was for ^•^OH, which is formed upon peroxynitrite self-decomposition (Scheme 1), where *k*(^•^OH + DMSO) = 6 × 10^9^ M^−1^ s^−1^ [24]. In the absence of CO_2,_ peroxynitrite decomposes to yield **^·^**OH and **^·^**NO_2_ radicals (Scheme 1), which oxidize hydroxylamine to RNO**^·^**. This oxidation is followed by multiple competing reactions of RNO**^·^** and of its oxidized form RN^+^=O, where in general *k*_5_ > k_7_ >> *k*_6_. All rate constants, previously determined for TEMPO-H (p*K*_1_ = 7.5) [14,15,25,26] are presented below. The protonated form RN^+^HOH is expected to be less reactive than RNO-H towards ^•^OH and ^•^NO_2_ radicals, and the values of *k*_5_ and *k*_6_ reflect the apparent rate constants at the pH studied.

^•^OH + RNO-H → RNO^•^ + H_2_O *k*_5_ = 1 × 10^9^ M^−1^ s^−1^ (pH 7.4) (5)

^•^NO_2_ + RNO-H → RNO^•^ + NO_2_^−^ + H^+^  *k*_6_ < 1 × 10^4^ M^−1^ s^−1^ (pH 6.8) (6)

^•^NO_2_ + RNO^•^ **^⇄^** RN^+^=O + NO_2_^−^ *k*_7_ = 7 × 10^8^ M^−1^ s^−1^ k_-7_ = 9 × 10^3^ M^−1^ s^−1^ (7)

RN^+^=O + ONOO^−^ → RNO^•^ + NO + O_2_  *k*_8_ = 6 × 10^6^ M^−1^ s^−1^ (8)

RN^+^=O + NO + OH^−^ → RNO^•^ + NO_2_^−^ + H^+^  *k*_9_ = 1 × 10^4^ M^−1^ s^−1^ (9)

Peroxynitrite self-decomposition yields ca. 30% ^•^OH and ^•^NO_2_ radicals, but due to multiple competing reactions 4–8, the determination of RNO^•^ by EPR spectroscopy cannot be used for quantitative assay of peroxynitrite in vitro. The situation is even more complicated under in vivo conditions where ^•^OH and ^•^NO_2_ are formed by other oxidizing reactions systems, such as Fenton-like reactions and peroxidase/H_2_O_2_/nitrite, respectively.

### 4.2. Superoxide Reaction with Hydroxylamines

Similar argumentation holds for the proposed use of hydroxylamines as monitors of superoxide, particularly in cases where the accumulated nitroxides are effective SOD-mimics. Seemingly, the accumulation of nitroxides, as end-products of hydroxylamines oxidation might serve for monitoring the superoxide flux. However, effective catalytic removal of superoxide radicals by nitroxides, particularly 6-membered ring nitroxides, totally pre-empts such an approach. It has been demonstrated that 6-membered ring nitroxides are better SOD-mimics compared to 5-membered ones, and that the SOD-activity decreases with the increase in the reduction potential of the nitroxide, *E*^o^(RN^+^=O/RNO^•^) [14,15]. Thus, since all hydroxylamine share similar rate constants of their reaction with superoxide at pH 7–8 (10^3^–10^4^ M^−1^ s^−1^ [9,10,11]), 3-CTPO-H is potentially a better spin probe than 3-CP-H for superoxide since *E*^o^(RN^+^=O/RNO^•^) = 1.0 V and 0.87 V, respectively [14,15], i.e., 3-CP-H is a better SOD-mimic.

## 5. Conclusions

The present study demonstrates that peroxynitrite does not react directly with cyclic hydroxylamines in the absence of CO_2_, and obviously in its presence, which is the predominant path of peroxynitrite decomposition in vivo. Peroxynitrite induces hydroxylamines oxidation to their respective nitroxides indirectly via reactions with the transient radical products of peroxynitrite self-decomposition, i.e., ^•^OH and ^•^NO_2_. However, the accumulated nitroxides are far below their expected yield, had the hydroxylamines fully scavenged all these radicals, due to multiple competing reactions of the oxidized forms of the hydroxylamines with ^•^NO_2_ and ONOO^−^. Hence, cyclic hydroxylamines cannot be used for quantitative assay of peroxynitrite in vitro. The situation is even more complex in vivo where ^•^OH/CO_3_^•−^ and ^•^NO_2_ are formed also by other oxidizing reactions systems, such as Fenton-like reactions and peroxidase/H_2_O_2_/nitrite, respectively.

The oxidation of cyclic hydroxylamines by superoxide yields their respective nitroxides, which are known as SOD-mimic. Therefore, the 5-membered ring hydroxylamines, which their respective nitroxides are poor SOD-mimics, might be considered as stoichiometric monitors of superoxide in vitro at highest possible concentrations and pH.

## Figures and Tables

**Table 1:** Nitroxide yield monitored upon reaction of ONOOH with RNO-H_2_^+^ at pH 4.3–4.6 (50 mM acetate buffer, 50 µM DTPA).

	[RN^+^HOH]_0_, mM	[ONOOH]_0_, µM	[RNO^•^], µM	Yield % ^a^
TEMPOL-H_2_^+^	5	118	5.2	4.4
	10	118	7.1	6.0
	50	118	9.7	8.2
TEMPONE-H_2_^+^	0.5	215	1.5	0.7
	2.5	215	16.7	7.8
TEMPO-H_2_^+^	10	122	40	32.8
3-CP-H_2_^+^	10	132	63.6	48.2

a—Oxidation yield expressed as100x[RNO**^·^**]/[ONOOH]_0_. The experimental error is ±10%.

**Table 2:** Initial rate (μM/min) of RNO^•^ accumulation upon irradiation of oxygenated solutions containing RNO-H, 0.1 M formate, 10 mM phosphate and 50 μM DTPA producing a flux of 4.0 ± 0.1 μM/min superoxide.

	pH 6.4	pH 7.4	pH 7.8
0.5 mM TEMPONE-H	1.4 ± 0.1	2.1 ± 0.1	2.6 ± 0.1
0.5 mM TEMPOL-H	1.1 ± 0.1	2.3 ± 0.1	3.0 ± 0.1
0.5 mM TEMPOL-H 20 μM TEMPOL		1.3 ± 0.1	
2 mM TEMPOL-H		2.4 ± 0.1	
0.5 mM 3-CP-H	2.2 ± 0.1	2.9 ± 0.1	3.1 ± 0.1
2 mM 3-CP-H			3.8 ± 0.2
2 mM 3-CP-H 50 μM 3-CP			3.8 ± 0.2
0.5 mM 3-CTPO-H			3.4 ± 0.1
1 mM 3-CTPO-H			4.0 ± 0.2

## Data Availability

Data is contained within the article.

## References

[1] Winterbourn C.C. (2020). Biological chemistry of superoxide radicals. ChemTexts.

[2] Bielski B.H.J., Cabelli D.E., Arudi R.L., Ross A.B. (1985). Reactivity of HO_2_/O_2_^−^ radicals in aqueous solution. J. Phys. Chem. Ref. Data.

[3] Szabó C., Ischiropoulos H., Radi R. (2007). Peroxynitrite: Biochemistry, pathophysiology and development of therapeutics. Nat. Rev. Drug Discov..

[4] Ferrer-Sueta G., Campolo N., Trujillo M., Bartesaghi S., Carballal S., Romero N., Alvarez B., Radi R. (2018). Biochemistry of peroxynitrite and protein tyrosine nitration. Chem. Rev..

[5] Zhang Y., Dai M., Yuan Z. (2018). Methods for the detection of reactive oxygen species. Anal. Methods.

[6] Dikalov S.I., Polienko Y.F., Kirilyuk I. (2018). Electron paramagnetic resonance measurements of reactive oxygen species by cyclic hydroxylamine spin probes. Antioxid. Redox Signal..

[7] Goldstein S., Lind J., Merenyi G. (2005). Chemistry of peroxynitrites as compared to peroxynitrates. Chem. Rev..

[8] Sampson J.B., Ye Y., Rosen H., Beckman J.S. (1998). Myeloperoxidase and horseradish peroxidase catalyze tyrosine nitration in proteins from nitrite and hydrogen peroxide. Arch. Biochem. Biophys..

[9] Dikalov S., Skatchkov M., Bassenge E. (1997). Quantification of peroxynitrite, superoxide, and peroxyl radicals by a new spin trap hydroxylamine 1-hydroxy-2,2,6,6-tetramethyl-4-oxo-piperidine. Biochem. Biophys. Res. Commun..

[10] Dikalov S., Skatchkov M., Bassenge E. (1997). Spin trapping of superoxide radicals and peroxynitrite by 1-hydroxy-3-carboxy-pyrrolidine and 1-hydroxy-2,2,6,6-tetramethyl-4-oxo-piperidine and the stability of corresponding nitroxyl radicals towards biological reductants. Biochem. Biophys. Res. Commun..

[11] Zhang R., Goldstein S., Samuni A. (1999). Kinetics of superoxide-induced exchange among nitroxide antioxidants and their oxidized and reduced forms. Free Radic. Biol. Med..

[12] Dikalov S., Grigor’ev I.A., Voinov M., Bassenge E. (1998). Detection of superoxide radicals and peroxynitrite by 1-hydroxy-4-phosphonooxy-2,2,6,6-tetramethylpiperidine: Quantification of extracellular superoxide radicals formation. Biochem. Biophys. Res. Commun..

[13] Samuni A., Goldstein S. (2020). Hydroxylamines inhibit tyrosine oxidation and nitration: The role of their respective nitroxide radicals. Free Radic. Biol. Med..

[14] Goldstein S., Merenyi G., Russo A., Samuni A. (2003). The role of oxoammonium cation in the SOD-mimic activity of cyclic nitroxides. J. Am. Chem. Soc..

[15] Goldstein S., Samuni A., Hideg K., Merenyi G. (2006). Structure-activity relationship of cyclic nitroxides as SOD mimics and scavengers of nitrogen dioxide and carbonate radicals. J. Phys. Chem. A.

[16] Saha A., Goldstein S., Cabelli D., Czapski G. (1998). Determination of optimal conditions for synthesis of peroxynitrite by mixing acidified hydrogen peroxide with nitrite. Free Radic. Biol. Med..

[17] Goldstein S., Michel C., Bors W., Saran M., Czapski G. (1988). A critical reevaluation of some assay methods for superoxide dismutase activity. Free Radic. Biol. Med..

[18] Goldstein S., Fridovich I., Czapski G. (2006). Kinetic properties of Cu,Zn-superoxide dismutase as a function of metal content-Order restored. Free Radic. Biol. Med..

[19] Beckman J.S., Beckman T.W., Chen J., Marshall P.A., Freeman B.A. (1990). Apparent hydroxyl radical production by peroxynitrite: Implications for endothelial injury from nitric oxide and superoxide. Proc. Natl. Acad. Sci. USA.

[20] Loegager T., Sehested K. (1993). Formation and decay of peroxynitrous acid: A pulse radiolysis study. J. Phys. Chem..

[21] Kissner R., Nauser T., Bugnon P., Lye P.G., Koppenol W.H. (1997). Formation and properties of peroxynitrite as studied by laser flash photolysis, high-pressure stopped-flow technique, and pulse radiolysis. Chem. Res. Toxicol..

[22] Kato Y., Shimizu Y., Yijing L., Unoura K., Utsumi H., Ogata T. (1995). Reversible half-wave potentials of reduction processes on nitroxide radicals. Electrochim. Acta.

[23] Israeli A., Patt M., Oron M., Samuni A., Kohen R., Goldstein S. (2005). Kinetics and mechanism of the comproportionation reaction between oxoammonium cation and hydroxylamine derived from cyclic nitroxides. Free Radic. Biol. Med..

[24] Mallard W.G., Ross A.B., Helman W.P. (1998). NIST Standard Reference Database. 40, Version 3.0.

[25] Samuni A., Goldstein S., Russo A., Mitchell J.B., Krishna M.C., Neta P. (2002). Kinetics and mechanism of hydroxyl radical and OH-adduct radical reactions with nitroxides and with their hydroxylamines. J. Am. Chem. Soc..

[26] Goldstein S., Samuni A., Russo A. (2003). Reaction of cyclic nitroxides with nitrogen dioxide:  The intermediacy of the oxoammonium cations. J. Am. Chem. Soc..

